# Partial, Rather than Full, BACE1 Inhibition May Be a Better Therapeutic Strategy for Alzheimer’s Disease Due to Effects of Complete Loss of BACE1 Activity on Adult Hippocampal Neurogenesis

**DOI:** 10.1523/ENEURO.0384-18.2018

**Published:** 2018-10-17

**Authors:** Rosalind S.E. Carney

## Abstract

**Highlighted Research Paper:** BACE1 Regulates Proliferation and Neuronal Differentiation of Newborn Cells in the Adult Hippocampus in Mice by, Zena K. Chatila, Eunhee Kim, Clara Berlé, Enjana Bylykbashi, Alexander Rompala, Mary K. Oram, Drew Gupta, Sang Su Kwak, Young Hye Kim, Doo Yeon Kim, Se Hoon Choi, and Rudolph E. Tanzi.

Alzheimer’s disease (AD) is thought to affect >5.5 million people in the United States and could be the third leading cause of death in older people (National Institute on Aging, 2016). One of the hallmarks of AD is the extracellular accumulation of β-amyloid protein and the formation of amyloid plaques. As β-site amyloid precursor protein cleaving enzyme 1 (BACE1) is an essential enzyme for the production of β-amyloid, BACE1 inhibition is currently being tested as a therapeutic strategy for AD in human clinical trials (Vassar, 2014). Supporting this approach is the observation that transgenic models of AD do not exhibit amyloid pathology when crossed with mice deficient in *BACE1* (Luo et al., 2003; Laird et al., 2005; McConlogue et al., 2007; Ohno et al., 2007).

The hippocampus is one of few brain regions in which neurogenesis persists in adulthood and enhancing adult hippocampal neurogenesis could potentially rescue some of the learning and memory defects observed in AD. Prior studies have shown that BACE1 does play a role in neurogenesis in early hippocampal development (Hu et al., 2013) and also regulates the proliferation and differentiation of neural precursor cells (NPCs) *in vitro* (Freude et al., 2011; Baratchi et al., 2012). However, it was unknown whether BACE1 has an endogenous function in the regulation of adult hippocampal neurogenesis. Therefore, in their *eNeuro* publication, Chatila et al., 2018, examined the proliferation, survival, and differentiation of newborn neurons in the dentate gyrus of mice deficient in *BACE1*.

Two-month-old wild-type (*BACE1*+/+), heterozygous (*BACE1*+/–) and homozygous *BACE1* knock-out (*BACE1*–/–) mice were injected with 5-bromo-2’-deoxyuridine (BrdU), a thymidine analog that is incorporated into replicating cells. The brains were harvested 1 d after injection. The number of BrdU-positive (BrdU+) cells was significantly higher in the dentate gyrus of *BACE1*–/– mice compared to both *BACE1*+/– and *BACE1*+/+ mice (Fig. 1). Increased proliferation in *BACE1*–/– mice only was also confirmed by immunolabeling with an anti-Ki67 antibody, a marker of cellular proliferation. These results indicate that complete, but not partial loss, of BACE1 activity increases cell proliferation in the dentate gyrus of young adult mice.

**Figure 1.**
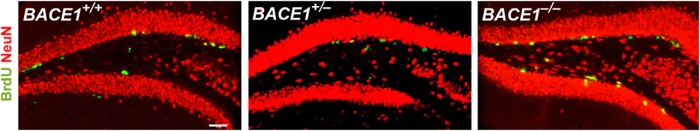
Complete loss of BACE1 activity increases NPC proliferation in the dentate gyrus. BrdU (green) and NeuN (red) coimmunofluoresence in the dentate gyrus of *BACE1*+/+, *BACE1*+/–, and *BACE1*–/– mice 1 d after BrdU injection. *BACE1*–/– mice exhibit significantly more BrdU+ cells compared to *BACE1*+/+ or *BACE1*+/– mice. Scale bar: 50 µm (adapted from Fig. 2*A* in Chatila et al., 2018).

Cell survival was similar in *BACE1*+/+, *BACE1*+/–, and *BACE1*–/– mice that received a single BrdU injection at two months of age and were killed four weeks later. Colabeling of these BrdU+ cells with neuronal, astrocytic, or oligodendrocytic markers was used to determine the fate of the increased number of NPCs that should be fated to differentiate into either granule neurons or glial cells. Compared to *BACE1*+/+ mice, fewer BrdU+ cells colocalized with the neuronal marker, NeuN, in the dentate gyrus of *BACE1*–/– mice. These observations indicate that complete loss of BACE1 activity impairs neuronal differentiation in the dentate gyrus.

The reduced number of differentiated neurons in the dentate gyrus of *BACE1*–/– mice was not countered by increased differentiation of NPCs into astrocytes or oligodendrocytes. Neither did complete loss of BACE1 activity affect neuronal maturation, as shown by double labeling of BrdU+ cells with doublecortin, a marker of immature neurons. Overall, these results show that complete, but not partial, loss of BACE1 activity affects adult hippocampal neurogenesis. *BACE1*–/– mice displayed increased NPC proliferation, but overall, these cells do not mature into neurons that could potentially compensate for the learning and memory defects associated with AD.

The MERCK Phase 3 clinical trial of the BACE1 inhibitor verubecestat was terminated due to adverse side effects. Chatila et al., 2018 findings suggest that partial, rather than full, BACE1 inhibition may have a higher therapeutic benefit to counter the neurodegenerative effects of AD. Partial inhibition of BACE1 could potentially reduce some of the adverse side effects observed with the use of verubecestat, including liver defects, weight loss, sleep disturbances, and suicidal thoughts.

Further studies in Professor Tanzi’s lab (Harvard Medical School) aim to determine the impact of BACE1 inhibitors on adult hippocampal neurogenesis in older mice and conditional knock-outs of *BACE1*. In the meantime, Chatila et al., 2018 findings suggest that maybe less is more regarding BACE1 inhibition and AD.
